# Supplementary material for: Assessing the role of the pelvic canal in supporting the gut in humans
Source: PLoS One. 2021 Oct 11;16(10):e0258341. doi: 10.1371/journal.pone.0258341 (PMC8504728; doi:10.1371/journal.pone.0258341)
Supplement: Attachment — Submitted filename: Response-To-Reviewers.docx [file pone.0258341.s001.docx]

Jeanelle Uy, Ph.D.

Department of Anthropology

California State University, Long Beach

[jeanelle.uy@csulb.edu](mailto:jeanelle.uy@csulb.edu)

May 12, 2021

Dear Dr. Rosenberg and Reviewers:

Thank you for the opportunity to revise and resubmit our manuscript. We think that the changes, though major, did not substantially change our original conclusions—the results still ­reflect differing support mechanisms in males and females. The female mechanism reduces their risk for pelvic floor disorders and the male mechanism focusing on spatial accommodation for the gut.

The following pages detail what has been changed in the manuscript for each section and the parentheses at the end of each bullet point indicate which reviewer(s) suggested the change. If there is no parenthetical note, then this change was made under our discretion. We also separate the changes into “major” and “minor” categories.

We sincerely appreciated the constructive reviews given, which shaped the study and the manuscript into a better paper. Thank you for your time in reviewing this revised manuscript.

Sincerely,

Jeanelle Uy, Ph.D.

Assistant Professor

**­**

**Introduction**

Minor

- Line 46: We cited Baragi et al. 2002 as one of the papers that suggest the pelvic canal morphology contributes to pelvic floor integrity (R3)
- Line 62: Wording was clarified regarding the description of the planes of the canal as “constricted” (R1)
- Line 66: A figure (Fig 1) showing the three planes studied was added for those readers who do not have the background knowledge of pelvic anatomy (R1)
- Line 75: The relationship of the gut with body weight was introduced here since we will be using weight as a covariate in the statistical analysis described later (R2)
- Line 87-91: We expand and clarify the explanation of pelvis development, including reference to Dunsworth’s 2020 paper (R1)
- Line 98: We modified the statement that originally erroneously indicated that the true pelvis’s “main role” was for visceral support (R1)

**Materials and Methods**

Major

- Line 124: We defined the clinical sample more thoroughly and added Table 1, which includes a summary of the sample’s body weight, height, and gut volume. Unfortunately, we did not have more background information about the participants. We address this in the added Limitations section in the Discussion that describes the information lacking from the sample used. (R2)
- Line 114: We clarified what is meant by “no observed abnormalities” from the original manuscript. (R1)
- Lines 140; 148-158: We reported the error analysis of our measurements and indicated which landmarks are “fuzzy” in Table 2. (R2)
- Line 144: We included a new measurement called S5 Position (θ_b_) which is a quantification of how far back the apex of S5 is from the ischial spines. Fig 2, which shows the landmarks, was updated to show θ_b_. A larger angle indicates an S5 that is farther away. (R1 and R2)
- Pubic symphysis width was excluded from the study because it could not be reliably measured and even when included, the results did not indicate it was significant when we modified the statistical approach; the other variables offer a richer discussion. (R1 had originally asked why this measure was used.)
- The paragraph describing why intestinal volume was used was moved to the Discussion, and integrated in a discussion considering the other abdominopelvic organs (R1)
- Line 188-205: The Statistical Analysis section was fully re-written.
  - We regressed canal measurements on gut volume (i.e., flipped the x and y axes) to better align with the pelvic floor hypothesis, which is that gut size explains pelvic variation, not the other way around (R1)
  - We included body weight as a covariate in our regression analyses (R2)
  - We adopted a more conservative alpha level (0.01) to reduce risk of Type I Error (R1 and R3)
  - We performed a post hoc analysis on the variables that were significant, to find the relative importance of GV vs. body mass in influencing R^2^ using the R package *relweights.* These are reported in Table 5.

Minor

- Line 140: The table including the landmark descriptions was added to the main text (Table 2), which was originally in the Supplementary Material. (R1)
- Instances of “gut volume” were replaced with “GV” for consistency
- Table 3, Fig 2: Medial projection of the ischial spines are now labeled with θ_a_.
- Line 136: Fig 2 was modified to reflect the removal of pubic symphysis width from the analysis. We removed the landmarks that were on the anterior superior public symphysis on the left and right pelvic bones.
- Tables and figures were moved to new positions in accordance with PLOS formatting requirements.

**Results**

Major

- We rewrote this entire section to reflect the results of the statistical reanalysis.
- For clarity, we reorganized this into three sections: Inlet, Midplane, and Outlet.
- We added two figures - Figs 3 and 4 - to reflect the new results.
- In the description of the results, we highlight the R^2^ values. (R1 and R2)
- We describe the relative importance of each predictor variable (GV or body mass) on R^2^.
- We also describe throughout any relevant differences between large-bodied and small-bodied people in the relationship of GV with the canal. (R2)
- Line 217: We added Table 5 to show the results of the *relweights* analysis described in the M&M section.

Minor

- We refrained from using the word “correlation” in describing our results because we did not specifically do a correlation analysis. Instead, we used “association” or “relationship”. (R1)

**Discussion**

Major

- Lines 290-295: We cite R^2^ values we found and added text to convey that we do not wish to over-interpret the results, especially with R^2^ values of 0.25 or lower. (R1 and R2)
- We removed all discussion of MML and pubic symphysis width since those were no longer relevant after the changes in analysis.
- Lines 330-346: We added a paragraph addressing the exclusion of other abdominopelvic organs in this study. We refer to Dunsworth’s (2020) paper here as well which suggested that uterus volume may influence pelvis size (inlet in particular). Since the size of the uterus cited in that paper is a bit larger than what more recent studies report, we also cite a more recent study that uses volumetric measurements from MRI which is more aligned with our CT method. (R1)
- Lines 362-380: We added a paragraph that situates our findings on the gut-canal relationship within the context of the many factors that influence pelvic canal variation. (R2)
- Lines 422-438: We added a Limitations section that addresses the shortcomings of our study sample (R2).

Minor

- Line 390: The trend of increasing prevalence of obesity in children is described in more neutral terms. The previous draft of this manuscript described “the increasing rate of excess weight”, however describing people as having “excess weight” can be subjective. We modified the phrasing to merely describe that body weight is increasing, which is more value neutral.
- Lines 397 and 437: We cited Baragi et al. 2002 in light of the limitations of our sample (we do not have ancestry or race info) and in the Implications subsection (R3)
- Lines 411-417: Some text was added to the “Clinical and Paleontological Implications” subsection to expand on the implications on paleoanthropology—particularly our assumptions about gut size using “pelvic types”. In light of how little variation is explained by gut size, it seems more prudent to refrain from predicting gut size using pelvic morphology of fossil remains.
