# Supplementary material for: Assessing the role of the pelvic canal in supporting the gut in humans
Source: PLoS One. 2021 Oct 11;16(10):e0258341. doi: 10.1371/journal.pone.0258341 (PMC8504728; doi:10.1371/journal.pone.0258341)
Supplement: Attachment — Submitted filename: Response-To-Reviewers.docx [file pone.0258341.s002.docx]

Jeanelle Uy, Ph.D.

Department of Anthropology

California State University, Long Beach

[jeanelle.uy@csulb.edu](mailto:jeanelle.uy@csulb.edu)

August 5, 2021

Dear Dr. Rosenberg and Reviewers:

We appreciated the kind and encouraging reviews. The following page details what has been changed in the manuscript for each section and the parentheses at the end of each bullet point indicate which reviewer(s) suggested the change. If there is no parenthetical note, then this change was made under our discretion.

Thank you once again for your time in reviewing this revised manuscript.

Sincerely,

Jeanelle Uy, Ph.D.

Assistant Professor

**­**

**Abstract**

- Line 23: Minor change in syntax for one sentence.

**Introduction**

- Line 45: We added a sentence acknowledging that locomotion may have also influenced the pelvic canal (Editor)
- Line 91: We changed the phrase “…the pelvis to adapt to a variety of effects” into something less vague (“factors such as birth, locomotion, and climate”) for clarity (R3)

**Materials and Methods**

- Line 161 Table 3 – we corrected the description for S5 Position (R3)
- Line 196: We provided a clearer explanation of how S5 projection was calculated (R3)

**Results**

- Line 240 Table 4: We modified this table so that the slopes for each predictor variable were reported along with their *p-*values. We removed the column reporting the intercept. (R1)
- We revised how the regression results were presented on Table 4 so that it appeared cleaner.
- Line 264-283:
  - We modified the Fig 3 caption to better describe the plot (R1)
  - We divided up Fig 3 into 3 separate figures (Figs 3, 4, and 6) to allow for the text to appear larger (Editor)
- Line 305-306: We noted that MAP in females and MML in males show significance when using an alpha of 0.05. (R1)

**Discussion**

- Line 347: We added a reference to the statement that there are sex differences in the occurrence of PFDs (R2)

**Other**

- We added one new citation (35) in the References section. (It is cited in Discussion.)
- We modified the in-text citations as necessary throughout.
- We corrected several grammatical errors (R3).
